# Patient-Centered Discharge and Postoperative Length of Hospital Stay in Primary Total Knee Arthroplasty

**DOI:** 10.7759/cureus.76271

**Published:** 2024-12-23

**Authors:** Yoshinori Ishii, Hideo Noguchi, Junko Sato, Ikuko Takahashi, Kei Ishii, Kai Ishii, Hana Ishii, Shin-ichi Toyabe

**Affiliations:** 1 Orthopaedic Surgery, Ishii Orthopaedic and Rehabilitation Clinic, Gyoda, JPN; 2 Breast Surgery, Ishii Orthopaedic and Rehabilitation Clinic, Gyoda, JPN; 3 Plastic and Reconstructive Surgery, Ishii Orthopaedic and Rehabilitation Clinic, Gyoda, JPN; 4 Division of Information Science and Biostatistics, Niigata University Hospital, Niigata, JPN

**Keywords:** american society of anesthesiologists physical status, healthcare costs, length of stay, patient-centered care, total knee arthroplasty

## Abstract

Background

This study aimed to identify factors affecting the length of hospital stay (LOS) after total knee arthroplasty (TKA) in patients classified as American Society of Anesthesiologists (ASA) physical status I or II, with a focus on patient-dependent determination of discharge. The goal was to explore strategies to shorten LOS.

Methods

A retrospective cohort study was conducted on 398 patients (494 knees) who underwent primary TKA. Factors associated with LOS were analyzed, including age, body mass index, operative time, clinical scores, anemia, albumin levels, age group, sex, and ASA physical status. Data are presented as medians (interquartile range).

Results

The median LOS for all patients was 35 days (range: 29-42 days). Patients aged 80 and over had a significantly longer LOS (39 days; range: 31-45) compared to younger age groups (vs. less than 60s: 32 days (range: 26-37 days), p=0.016; vs. 60s: 33 days (range: 30-38 days), p<0.001; vs. 70s: 35 days (range: 29-43 days), p=0.033). Females also had a significantly longer LOS (36 days [range: 30-43 days]) compared to males (31 days [range: 24-40 days]; p<0.001). Multivariate analysis confirmed that being aged 80 or over (p<0.001) and being female (p<0.001) were significantly associated with longer LOS.

Conclusion

Postoperative interventions tailored for patients aged 80 years or older and females may be effective in shortening LOS after TKA. A patient-centered approach suggests a maximum LOS of approximately five weeks. Balancing provider-driven early discharge with patient-centered extended stays is crucial for optimizing postoperative care.

## Introduction

With the aging population, the global prevalence of osteoarthritis (OA) has increased, placing a significant burden on healthcare systems. Total knee arthroplasty (TKA) is a common surgical procedure that alleviates pain and improves joint function in patients with end-stage OA [1]. Recently, TKA has been increasingly performed in older patients, including those in their 80s and 90s [2-4]. Consequently, the demand for TKA is projected to continue rising [5].

While the increasing demand for TKA raises concerns about escalating healthcare costs [6], TKA can significantly improve patients' quality of life by alleviating pain, enhancing physical function, and improving activities of daily living (ADLs) [7]. Additionally, improvements in ADLs have been associated with increased bone quality [8] and bone mineral density [9], reducing the deterioration of arteriosclerosis [10]. These positive effects may contribute to reduced healthcare costs by decreasing the need for medications such as bone-density-building drugs and anti-arteriosclerosis agents.

Length of hospital stay (LOS) post-TKA is a major component of healthcare costs [11]. Prolonged hospitalization can strain healthcare budgets. In Japan, small-scale hospitals (less than 20 beds) with inpatient facilities and operating rooms are suitable for treating less severe patients. Due to lower government-regulated inpatient costs compared to larger hospitals, these facilities have the potential to reduce TKA-related healthcare costs. However, the Japanese healthcare system allows patients to determine their LOS to a certain extent, which contrasts with Western healthcare systems where economic factors strongly influence LOS decisions.

While patients with American Society of Anesthesiologists (ASA) physical status classifications [12] III and IV have traditionally been considered unsuitable for treatment in small-scale hospitals depending on the patient's condition and the healthcare facility's capacity, these patients may also be considered candidates for treatment in such settings in Japan. However, strict admission criteria and appropriate staffing are essential to ensure patient safety. Optimizing LOS post-TKA can not only reduce healthcare costs but also improve patients' quality of life.

Therefore, healthcare providers, patients, and payers should collaborate to establish evidence-based criteria for LOS and strengthen discharge support systems. The objective of this study was to identify factors influencing the postoperative LOS following primary TKA in patients with ASA physical status classifications I and II, where patients determined their discharge criteria. The goal was to identify intervention points to shorten LOS.

## Materials and methods

A total of 398 patients (494 knees) who underwent primary TKA between July 2002 and October 2024 were included in this study (Table 1). All patients underwent surgery using the New Jersey LCS total knee system (DePuy, Warsaw, IN) for primary OA. Patients with severe comorbidities and those classified as ASA physical status III or IV were excluded. As a general rule, patients who desired bilateral knee replacements underwent staged procedures. However, one patient who underwent bilateral TKA during the same hospitalization and two patients who required revision surgery due to deep infection were excluded. This study did not include patients who underwent revision TKA or conversion from high tibial osteotomy. The local institutional review board approved this study (ID number: 2024-10), and all patients provided informed consent.

**Table 1:** Patients’ background M, male; F, female; BH, body height; BW, body weight; BMI, body mass index; ASA, American Society of Anesthesiologists; HSS, hospital for special surgery; RBC, red blood cell count; Hb, hemoglobin; Ht, hematocrit Values are expressed as n or median (25th percentile, 75th percentile).

Variables	Median (interquartile range)	Range
M (knees):F (knees)	78 (65):416 (333)	N/A
Age (year)	74 (69-78)	34-90
BH (cm)	151 (146-155)	135-184
BW (kg)	58 (53-66)	38-130
BMI (kg/m²)	26 (23-28)	18-42
Operation time (minutes)	55 (50-62)	39-116
ASA status (I, II) [12]	74/ 420	N/A
HSS score [13]	45 (37-51)	10-75
RBC (x10⁴/mL)	430 (406-454)	310-586
Hb (g/dL)	13.1 (12.3-13.9)	8.7-18.2
Ht (%)	40.3 (38.3-42.7)	28.1-53.2
Serum Albumin (g/dL)	4.3 (4.2-4.5)	2.3-5.1

Perioperative protocol

A standardized surgical technique was employed for all patients, involving a midline incision with medial parapatellar arthrotomy and routine patellar eversion under tourniquet control. The incision was extended medially to the level of the semimembranosus tendon insertion to address soft tissue contractures and achieve optimal soft tissue balance. Osteophytes were removed as needed. Patellar resurfacing and lateral retinaculum release were not performed.

Postoperatively, patients were managed with a compression dressing and intra-articular drain. Full weight-bearing with a cane was permitted on the first postoperative day under physical therapy supervision. A comprehensive rehabilitation program, including isometric and active-assisted range-of-motion exercises, quadriceps and hamstring strengthening, and gait training, was initiated. Perioperative antibiotics and analgesics were administered, but thromboprophylaxis was not routinely used. A combination of diclofenac sodium (50 mg suppository), ketoprofen (15 mg IM), and pentazocine (15 mg IM) was used for postoperative analgesia, tailored to each patient's needs. Diclofenac sodium and ketoprofen are nonsteroidal anti-inflammatory drugs that inhibit prostaglandin production at nociceptors. Pentazocine is a non-narcotic analgesic that suppresses central nervous system conduction. We did not employ multimodal analgesics such as peripheral nerve blocks or periarticular infiltration analgesia, as these techniques can be associated with limitations such as an increased risk of falls, delayed rehabilitation after nerve blocks, and variable outcomes due to infiltration techniques in periarticular infiltration analgesia.

This retrospective cohort study, using medical records, aimed to investigate factors influencing LOS in patients undergoing primary TKA. The subjects were 34- to 90-year-old patients who underwent TKA, categorized into four age groups: 34-59 years (under 60s), 60- 69 years (60s), 70-79 years (70s), and 80-90 years (80s). The following variables were examined for their association with LOS: age [14-19], body mass index (BMI) [20-22], operative time [14,23,24], and hospital for special surgery (HSS) knee score [13] as knee clinical score [16]; preoperative degree of anemia [15,18,21,24-26] such as hemoglobin (Hb), hematocrit (Ht), and red blood cell count (RBC), and serum albumin level [23] as continuous variables; and age group [21,22,25,27,28], sex [14-19,21-23,25,27-30], and ASA physical status [14,17,18,21-24] as categorical variables. These variables have been suggested in previous studies to potentially influence LOS in TKA patients. The relationship between each variable and LOS was analyzed using the statistical method. Additionally, multivariate analysis was performed to consider multiple variables simultaneously.

Discharge criteria

LOS by discharge to home was determined through negotiation with surgeons, rehabilitation staff, and patients and their families. In such a situation, patient satisfaction and confidence in daily life after TKA were prioritized. There have been no medically induced discharges in this series, although we allow the patients to be discharged from the clinic with physical therapist’s confirmation of independent cane walking up and down stairs, according to clinical pathways provided before TKA.

Statistical analysis

Because some variables did not show a normal distribution as determined by the Kolmogorov-Smirnov and Shapiro-Wilk’s test, or the Q-Q plot, these variables were analyzed using a nonparametric method. For comparisons of continuous variables, we used the Mann-Whitney U test for independent samples and the Kruskal-Wallis test. Bonferroni's method was used to correct for multiple comparisons. Spearman’s rank correlation coefficient was employed to assess relationships between two variables. Correlation strengths were categorized as follows: strong (0.70- 1.0), moderate (0.40- 0.69), and weak (0.20- 0.39). The factors associated with LOS were examined by multiple regression analysis. The candidate independent variables entered into the multiple regression analysis were age, BMI, operative time, HSS score, RBC, Hb, Ht, serum albumin, age group, sex, and ASA grade. All statistical analyses were performed using R Version 4.4.1 (R Foundation for Statistical Computing, Vienna, Austria). Values were presented as medians (interquartile range), and a p-value of <0.05 was considered statistically significant.

## Results

Post-hoc power analysis revealed that the Mann-Whitney U test had 95% power, Spearman's correlation had 96% power, and multiple regression had 36% power, all at a significance level of 0.05. The median LOS was 35 days (range: 29-42 days). To identify factors influencing LOS, we analyzed patient background factors. No significant associations were found with continuous variables (Table 2).

**Table 2:** Univariate analyses (I): correlations between length of hospital stay and the study variables by Spearman’s rank correlation coefficient p-value, probably value; r, correlation coefficient; BMI, body mass index; HSS, hospital for special surgery; RBC, red blood cell count; Hb, hemoglobin; Ht, hematocrit

Variables (N=494)	vs. Length of hospital stay	
	p-Values	r
Age (years)	0.171	0.181
BMI (kg/m²)	-0.037	0.418
operative time (min.)	-0.164	<0.001
HSS score [13]	-0.041	0.359
RBC (x10⁴/mL)	-0.073	0.104
Hb (g/dL)	-0.078	0.085
Ht (%)	-0.076	0.092
Serum Albumin (g/dL)	-0.008	0.853

However, significant differences were observed between the categorical variables of age and sex. First, the Kruskal-Wallis test revealed a significant difference among the four age groups (p=0.002). Post-hoc analysis using Bonferroni correction showed that patients aged 80 and over had significantly longer LOS (39 days; range: 31-45 days) compared to other age groups (vs. less than 60s [32 days, range: 26-37 days, p= 0.016], vs. 60s [33 days, range: 30-38 days, p<0.001], vs. 70s [35 days, range: 29-43 days, p=0.033]) (Table 3).

**Table 3:** Univariate analyses (II): comparison between the factors and length of clinic admission (LOS) for discrete variables analyzed using the Mann–Whitney U test and the Kruskal-Wallis test Less than 60s: 34 to 59 years old; 60s: 60 to 69 years old; 70s: 70 to 80 years old; over 80s: 80 to 90 years old Values are expressed as n or median (25th percentile, 75th percentile). ASA, American Society of Anesthesiologists; LOS, length of hospital stay

Variables (N=494)	LOS (all), 35 days (range: 29-42 days)					
Age group	Less than 60s (N=19)	60s (N=108)		70s (N=274)		Over 80s (N=93)
LOS, days (range)	32 (26-37)	33 (30-38)		35 (29-43)		39 (31-45)
Less than 60s	N/A	0.472		0.304		0.016
60s	0.472	N/A		0.249		<0.001
70s	0.304	0.249		N/A		0.033
Over 80s	0.016	<0.001		0.033		N/A
Variables	LOS (days)		LOS (days)			p-Value
Sex	Male (78)	31 (24-40)	Female (416)		36 (30-43)	<0.001
ASA (knees) [12]	I (74)	31 (28-45)	II (420)		35 (30-42)	0.286

Regarding sex, females also had a significantly longer LOS (36 days; range: 30-43 days) compared to males (31 days; range: 24-40 days) (p<0.001) (Table 3). Multivariate analysis confirmed that being aged 80 (p<0.001) or over and being female were significantly associated with longer LOS (p<0.001) (Table 4).

**Table 4:** Multiple regression analysis S.E., Standard error; Sig., significance; CI, confidence interval

	B	S.E.	Β	Sig.	95% CI	
(Constant)	30.770	1.443		<0.001	28.39	33.15
Over 80s	6.390	1.429	0.196	<0.001	4.03	8.75
Sex (male 0, female 1）	6.030	1.524	0.174	<0.001	3.52	8.54

## Discussion

Two key findings emerged from this study. First, in line with previous reports, our analysis of Japanese patients, who were ASA I or II without severe comorbidities and could determine their discharge date in consultation with their physician, revealed that females and patients aged 80 years or older tended to have longer hospital stays. Second, in contrast to recent reports from Western and Asian countries showing a trend toward shorter hospital stays, our results demonstrated no significant reduction in LOS compared to a decade ago, with an average LOS of approximately five weeks.

While some studies [25] have suggested longer hospital stays for male patients, a substantial body of research [14-19,21-23,27-30] has consistently identified female sex as a risk factor for prolonged LOS. Our findings corroborate these latter studies, confirming that female patients tend to have longer hospitalizations. Regarding age, we observed no significant differences in LOS for patients under 80 years of age. This contrasts with previous studies [25, 27] that reported significant age-related differences around 70 years of age. However, our analysis revealed a significant increase in LOS for patients aged 80 and older. When focusing on lower-risk patients (ASA I and II), age appears to have a minimal impact on LOS up to 70 years of age.

Meanwhile, no correlations were found between LOS and other previously reported factors. This may be attributed to the fact that our study population was limited to ASA I and II patients without severe comorbidities, and all surgeries were performed by a single experienced surgeon using the same surgical technique. Regarding the latter, the minimal variation in operative time due to the consistency of the surgeon's technique may have limited the impact of operative time on LOS.

A significant variation in postoperative hospitalization duration following TKA has been observed across different countries, including Asia and Europe, from 2006 to 2012. For instance, the average LOS ranged from 3.8 days in the United States [31] to 35.1 days in Japan [28], with intermediate values reported for the Netherlands (6.2-7.5 days) [32], Germany (median 10 days) [33], Taiwan (8.66 ± 2.92- 10.79 ± 3.74 days) [34], and France (24.1 ± 8.1 days) [29]. These disparities are likely attributed to differences in healthcare systems across countries. While Western countries tend to have shorter LOS due to provider-driven initiatives and economic factors, Japan has a system that allows patients to determine their LOS based on reasonable grounds such as pain or mobility difficulties, leading to longer hospitalizations. In recent years, Western countries have reported an increasing trend toward shorter hospitalizations, with some even offering same-day surgery [35,36] or hospitalization within three days [14,17,24]. Although Japan has also experienced a decline in LOS, from an average of 35-54 days [28,37] more than a decade ago to 21-24 days [38,39] in recent years, it still remains longer compared to Western countries. This discrepancy is likely influenced by a complex interplay of factors, including Japan's unique healthcare system, patient expectations, and provider attitudes.

Our study found that the postoperative hospitalization period following TKA was approximately five weeks, aligning with previous reports in Japan [28,37]. Despite implementing an Enhanced Recovery After Surgery protocol with early rehabilitation, our institution's discharge criteria are based on the patient's confidence in managing at home and the family's readiness, often resulting in longer hospital stays. In contrast, Western countries, driven by economic considerations, have been promoting earlier discharge, leading to shorter hospitalization periods. However, premature discharge may compromise patient quality of life if functional recovery is inadequate. Healy et al. have argued that the pursuit of economic benefits should not come at the expense of patient care [40]. Optimizing the LOS following TKA is a critical issue that requires balancing patient satisfaction with healthcare costs. As Shah et al. pointed out, TKA treatment should aim to minimize hospitalization while maximizing functional recovery [22]. To achieve this goal, it is essential to establish optimal hospitalization periods that consider the healthcare systems and patient characteristics of each country. Orthopedic surgeons must continue to strive for better patient care while navigating the complex balance between patient satisfaction and healthcare economics.

This study is a retrospective chart review and has several limitations, including disparity in the number of male and female patients and the restriction to ASA I and II patients. However, considering that TKA is a procedure aimed at improving function, the results of this study provide valuable insights into a patient population considered appropriate for TKA. By minimizing biases related to surgeon and surgical technique, we were able to more accurately evaluate the impact of patient factors on LOS. However, given the 20-year duration of this study, it is important to acknowledge potential confounding from unmeasured factors such as changes in lifestyle and socioenvironmental conditions.

## Conclusions

Our study demonstrates that postoperative interventions may significantly reduce LOS for females aged 80 and older following TKA in patients with ASA I and II knee OA. Our findings suggest that this patient cohort experiences the longest LOS among reported studies, providing crucial implications for the development of sustainable healthcare systems worldwide. To optimize postoperative care in each country, a balanced approach is crucial. This involves considering a spectrum of care options, ranging from provider-driven same-day discharges to patient-centered, longer hospitalizations.
